# NEUROD1 mutation in an Italian patient with maturity onset diabetes of the young 6: a case report

**DOI:** 10.1186/s12902-021-00864-w

**Published:** 2021-10-15

**Authors:** Lucia Brodosi, Bianca Baracco, Vilma Mantovani, Loris Pironi

**Affiliations:** 1 IRCCS Azienda Ospedaliero-Universitaria di Bologna, Via Albertoni, 15, I-40138 Bologna, Italy; 2 University of Bologna, Bologna, Italy

**Keywords:** Case report. MODY 6. NEUROD1. Hyperglycaemia. Sulphonylureas. Cardiomyopathy

## Abstract

**Background:**

Maturity Onset Diabetes of the Young (MODY) is a monogenic, autosomal, dominant disease that results in beta-cells dysfunction with consequent hyperglycaemia. It represents a rare form of diabetes (1–2% of all the cases).

Sulphonylureas (SUs) represent the first-line treatment for this form of diabetes mellitus.

NEUROD1 is expressed by the nervous and the pancreatic tissues, and it is necessary for the proper development of beta cells. A neurogenic differentiation factor 1 (NEUROD1) gene mutation causes beta-cells dysfunction, inadequate insulin secretion, and hyperglycaemia (MODY 6).

**Case presentation:**

We have documented a new missense mutation (p.Met114Leu c.340A > C) of the NEUROD1 gene, pathogenetic for diabetes mellitus, in a 48 years-old man affected by diabetes since the age of 25 and treated with insulin basal-bolus therapy. Unfortunately, an attempt to replace rapid insulin with dapagliflozin has failed. However, after the genetic diagnosis of MODY6 and treatment with SUs, he was otherwise able to suspend rapid insulin and close glucose monitoring. Interestingly, our patient had an early onset dilated cardiomyopathy, though no data about cardiac diseases in patients with MODY 6 are available.

**Conclusions:**

Diagnostic criteria for MODY can overlap with other kinds of diabetes and most cases of genetic diabetes are still misdiagnosed as diabetes type 1 or 2. We encourage to suspect this disease in patients with a strong family history of diabetes, normal BMI, early-onset, and no autoimmunity. The appropriate therapy simplifies disease management and improves the quality of the patient’s life.

## Background

Maturity Onset Diabetes of the Young (MODY) is a genetic form of diabetes caused by a single gene mutation that impairs beta-cell function and development [1, 2]. It has peculiar clinical features as impaired insulin secretion, early hyperglycaemia onset, and negative beta-cells antibodies [3, 4]. MODY represents 1–2% of all cases of diabetes, but this condition is often underdiagnosed or misdiagnosed as diabetes type 1 or 2 because of some similar clinical features. Especially in adults, the clinical diagnosis is still very challenging [5].

Thanks to new genetic techniques, some of the molecular mechanisms that lead to MODY have been cleared up in the last few years. More specifically, at least fourteen different genes are responsible for MODY, and many mutations have been identified [6].

Like many genetic disorders, MODY is transmitted in an autosomal dominant mode. Each form of MODY is characterized by unique genetic, clinical, and metabolic features according to the tissues in which the responsible genes are expressed. The most common types are caused by mutations in ´ hepatocyte nuclear factor 4 alpha (HNF4A), Glucokinase (GCK), and hepatocyte nuclear factor 1 alpha (HNF1A) genes [7].

Although a few case-reports of MODY6 were published in the last few years, this form of diabetes remains very rare, and many details about the phenotypic tracts and the correct pharmacological approach of the disease still have to be investigated. We have documented a new missense mutation (p.Met114Leu c.340A > C) of the Neurogenic differentiation factor 1 gene (NEUROD1), pathogenetic for diabetes mellitus. Interestingly, our patient had an early onset dilated cardiomyopathy, though no data about cardiac diseases in patients with MODY 6 are available.

As this variant is not present in population databases (GnomAD exomes and genomes), we cannot exclude that it could arise from a common ancestor, or in the alternative, it could be a mutational hot spot.

## Case presentation

**Fig. 1:**
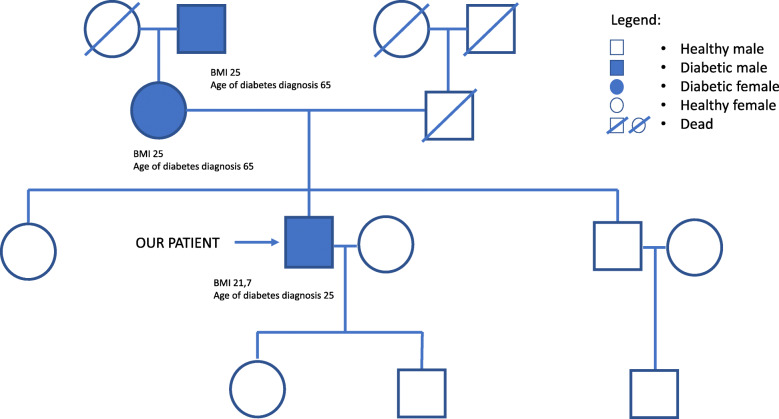
Patient’s family tree

We want to report the case of a 48 years old man diagnosed with type 2 diabetes mellitus from the age of 25. His prior medical history included a dilated cardiomyopathy treated with a pacemaker at the age of 42. His mother and maternal grandfather had been diagnosed with type 2 diabetes mellitus, while his father was dead for natural causes and he was not affected by diabetes. In addition, his brother and sister were healthy, with no sign of hyperglycaemia. He also had two children in apparently good health (Fig. 1).

**Fig. 2:**
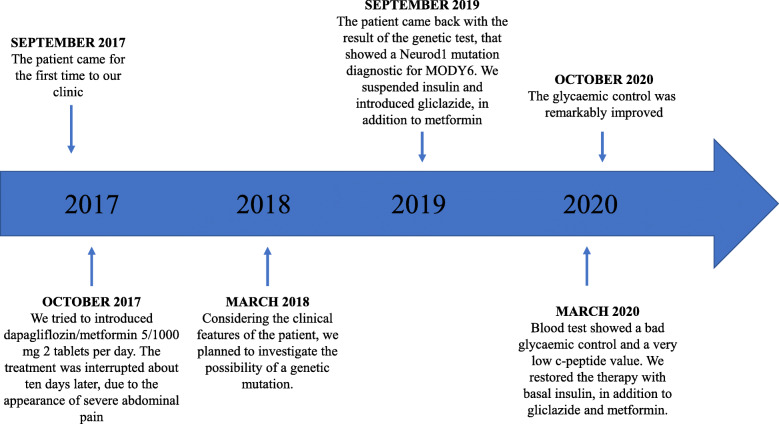
Graphic timeline

The patient came for the first time to our clinic in September 2017. He had no diabetic complications (nephropathy, neuropathy, or retinopathy) and did not report any diabetic signs or symptoms. He was following a regular treatment for type 2 diabetes with basal-bolus insulin associated with metformin (Table 1, Visit 1 and Table 2, Visit 1), set during a previous visit to another clinic. Unfortunately, no HOMA-IR was available to detect if the patient was affected by insulin resistance. In addition, the insulin dosage required for the calculation would be affected by exogenous insulin therapy. The patient’s clinical features, blood parameters, and therapy at his first medical control are summarized in Tables 1 and 2, respectively. For easier visualization of the case, look at Fig. 2.

**Table 1:** Patient’s clinical features and blood parameters over time

	September 2017	March 2020	October 2020
Arterial blood pressure systolic/diastolic (mmgHg)	134/78	110/70	120/80
Body weight (kg)	68	66	67
BMI (kg/m^2^)	21,7	21,1	21,4
Glycated hemoglobin (%)	8,6	10,7	7,5
Fructosamine (micromol/L)	341	missing data	264
Fasting blood glucose (mg/dL)	111	288	83
C-peptide (ng/mL)	0.4	0.8	0.6
Creatinine (mg/dL)	0,73	0,64	0,74
VFG (ml/min)	108	114	107
GOT (UI)	13	17	21
GPT (UI)	19	31	30
Total cholesterol (mg/dL)	140	115	105
Triglycerides (mg/dL)	72	71	79
LDL cholesterol (mg/dL)	74,2	47	38
HDL cholesterol (mg/dL)	52	54	51
Microalbuminuria (mg/L)	15	< 5	10
Urinary ketones	not detected	not detected	not detected

Fructosamine normal range (118–282 micromol/L); C-peptide normal range (0.9–7.1 ng/mL).

**Table 2:** Patient’s therapy over time

	September 2017	March 2020	October 2020
Insulin glargine 100 U/ml	18–22 UI at 10.00 pm	.	16–18 UI at 10.00 pm
Insulin lispro 100 U/ml	5 UI before breakfast, 6 UI before lunch and 6 UI before dinner	.	.
Metformin	500 mg tid	=	=
Gliclazide 60 mg RM	.	1 tablet od	1 tablet od
Bisoprolol	5 mg od + 10 mg od	=	=
Ramipril	10 mg od	=	=
Simvastatin/ezetimibe	10/10 od (poor compliance)	10/10 od (good compliance)	=
Lansoprazole	15 mg od	=	=

=: continued without modification.

Due to the poor glycaemic control, we scheduled a medical examination after a month, asking the patient to monitor capillary blood glucose at home in the morning and before every meal, writing the values in a diary. We also required the detection of beta-cell antibodies to investigate the possibility of an autoimmune etiology of diabetes.

When the patient came back to the outpatient clinic in October 2017, blood test results were not significant for autoimmune diabetes, and the values of capillary blood glucose at home showed an acceptable trend of diabetes (most of them were between 90 and 130 mg/dL in the morning and before meals). Therefore, according to the reasonable glycemic control, we modified the patient’s treatment by removing Lispro insulin before meals and introducing an oral hypoglycemic agent of the SGLT-2 inhibitors group (dapagliflozin) associated with metformin (dapagliflozin/metformin 5/1000 mg 2 tablets per day). However, the patient interrupted the treatment with dapagliflozin/metformin about ten days later due to the appearance of severe abdominal pain that spontaneously resolved at the suspension of the therapy. He, therefore, continued treatment with only metformin (500 mg 3 tablets per day) and insulin glargine, maintaining still excellent values (90–130 mg/dL) despite the reduction of the pharmacological therapy. In March 2018, the glycated hemoglobin had remained high at an updated blood test.

Considering the clinical features of the patient, we planned to investigate the possibility of a genetic mutation.

Targeted Next Generation Sequencing (NGS) of the 14 MODY genes, as well as Wolfram syndrome 1 gene (*WFS1)* and insulin receptor gene (*INSR)*, was performed by using amplicon-based libraries and Ion Gene Studio System S5, according to the manufacturer’s instructions (Thermo Fisher Scientific Inc.). The variant classification was performed according to the American College of Medical Genetics and Genomics (ACMG) standards by using the VarSomeClinical platform (https://varsome.com) [8]. Possible pathogenic variants were confirmed by Sanger sequencing. Large rearrangements in MODY1, 2, 3, and 5 genes were excluded by multiplex ligation-dependent probe amplification (MLPA) (MRC-Holland, Amsterdam, NL).

In September 2019, the genetic test showed heterozygosity for the missense variant p.Met114Leu c.340A > C in *NEUROD1* (NM_002500.4)*,* linked to MODY6. The variant was not found in GnomAD exomes and genomes and resulted in pathogenic prediction from 10 computational analyses (DEOGEN2, EIGEN, M-CAP, MVP, MutationAssessor, MutationTaster, PrimateAI, REVEL, PolyPhen-2, and SIFT) versus two benign predictions (DANN and FATHMM-MKL).

At the time of the molecular diagnosis, this variant was previously undescribed, and we classified it as a variant of uncertain significance (VUS). Only recently, Bouillet et al.described the same *NEUROD1* variant associated with MODY6 in a French family and classified it as a mutation [9].

International guidelines report sulphonylureas as the first-line treatment for monogenic diabetes, so we decided to start therapy with gliclazide RM (60 mg) suspending insulin at all (Table 2, Visit 2).

Unfortunately, glycaemic values did not benefit from the treatment with sulphonylureas, and at the following medical examination in March 2020, the patient showed inadequate glycaemic control and a very low c-peptide value (Table 1, Visit 2). We, therefore, restored the therapy with basal insulin, in addition to gliclazide and metformin. Metformin was maintained to provide a better insulin sensitivity and because of its cardiovascular protective effect. In October 2020, the glycaemic control was remarkably improved with this kind of scheme therapy (Table 1, Visit 3 and Table 2, Visit 3), practicing only one injection a day and without the necessity to measure blood sugar after every meal.

He referred us that his quality of life has remarkably improved, although this data was not measured with specific questionnaires. Two and a half years have passed from the first visit to obtaining a glycaemic compensation with stable therapy. During the follow-up, no hypoglycaemia was detected, and the patient did not develop any diabetic complication.

Since MODY is characterized by autosomal dominant inheritance, the family’s counseling was provided to the patient. His brother and sister did not show hyperglycaemia, either evidence of *NEUROD1* mutation at the genetic testing. The patient’s mother had been diagnosed with type 2 diabetes mellitus for many years but, because of her old age and immobility, she refused to come to our outpatient clinic for genetic testing. The patient signed written informed consent for the publication of his laboratory and clinical data.

## Discussion and conclusions

Monogenic diabetes should be suspected in non-obese young patients with no insulin-resistance, strong family history of diabetes, and negative beta-cell antibodies (even if a few cases of MODY with positive autoimmunity are reported) [10, 11].

Nowadays, Next Generation Sequencing (NGS) allows the simultaneous sequencing of the genes linked to MODY. In this way, very rapid results are obtained at a lower cost [12–14]. However, even today, genetic testing is not affordable in many hospitals all over the world, and it should be requested only in case of a strong clinical suspect [15]. Genetic testing and the detection of the mutation at the basis of the disease are necessary to provide the proper prognostic information and set up the best clinical management and pharmacological therapy for the patient [16, 17]. Besides, family counseling should be provided for all patients diagnosed with MODY because of the autosomal dominant inheritance that characterizes this condition [18].

The therapeutic approach differs among the various forms of MODY according to their clinical characteristics and molecular causes. In some cases, insulin administration is necessary to obtain better glucose control, but some patients affected by MODY benefit from oral hypoglycemic agents (e.g., sulphonylureas) without using insulin [19]. The cause of this phenotypical heterogeneity is not clear yet, but it can be suspected that not only genetic but also environmental parameters play an important role in the trend of diabetic illness. The therapy aims to improve the quality of life and prevent the complications of diabetes [20].

*NEUROD1* encodes a member of the NeuroD family of basic helix-loop-helix (bHLH) transcription factors. The protein forms heterodimers with other bHLH proteins and activates transcription of genes that contain a specific DNA sequence known as the E-box. This transcription factor is expressed by neuronal and pancreatic endocrine cells, and it plays a crucial role in the normal development and maintenance of these tissues. More specifically, in the pancreatic tissue, it is involved in the regulation of insulin synthesis and secretion [19].

It is also reported that the *NEUROD1* gene is involved in the transactivation of the sulphonylurea receptor 1 gene [20].

Recent evidence has proven that the inactivation of the *NEUROD1* gene in human embryonic stem cells severely impairs their differentiation from pancreatic progenitors into insulin expressing cells and that a genetic mutation of the *NEUROD1* gene leads to hyperglycaemia and diabetes (MODY6) [21].

The disease usually occurs at the age of 20–25 in patients with a heterozygous mutation and often shows a familiar distribution. The transmission pattern from the mother is more often reported than that from the father, and it might contribute to an early onset of the disease, probably due to the exposure to elevated blood glucose levels during pregnancy. These patients usually present a mild form of diabetes and are treated with oral hypoglycemic agents and no insulin. Very few cases of a homozygous mutation have been reported, and this kind of condition usually leads to neonatal diabetes [20].

Recent findings have also shown a connection between *NEUROD1* gene mutation and central nervous system abnormalities. More specifically, *NEUROD1* expression is necessary for the correct development and function of the cerebellum, inner ear, and retina and an impairment of the gene can cause mental disability, hippocampal hypoplasia, hearing loss, and epilepsy. It is reported that such abnormalities usually affect only patients with the homozygous mutation, but some recent evidence suggests that they can occur even in heterozygous patients [20].

In this case-report we reported the description of a heterozygous mutation of *NEUROD1* gene (p.Met114Leu c.340A > C) in a 48 years-old man wrongly diagnosed with type 2 diabetes. The same genetic mutation was recently detected in three members of a French family, confirming the association to MODY6 [9]. Like our patient, the French family members displayed the typical clinical characteristics of MODY: onset before age 25 years, insulin independence at the beginning of the disease, absence of β-cell autoimmunity and a two generation family history of diabetes. While French patients developed proteinuria, retinopathy and coronary heart disease, no diabetic complication was detected in our patient.

Data about MODY 6 are still lacking, and further studies are needed to identify a more significant number of cases and point out the most important clinical features that characterize the disease. In particular, there are no data in the literature that show a specific association between *NEUROD1* mutation and cardiomyopathy, so we couldn’t clarify if our patient developed the cardiac disease as a direct consequence of his genetic modification, because of the diabetic condition or the two pathologies are independent each other. In the literature, we found only one study investigating the link between MODY and heart disease. However, it aimed to estimate cardiovascular risk by coronary artery calcification (CAC) score in 29 patients affected by MODY caused by glucokinase (GCK) mutations [22]. The authors found that the CAC score in GCK-MODY was similar to control individuals from the same family and household and is significantly lower than type 2 diabetes. Thus, from our point of view, this study is irrelevant for our case, and future works are necessary to clarify a possible connection between MODY6 and cardiac disease. MODY diabetes is often misdiagnosed as diabetes type 1 or 2. In Turkey, 230 children with atypical presentations for T1DM and T2DM were tested for mutations in the following genes: *GCK-HNF1A-HNF4A-HNF1B-PDX1-NEUROD1-KLF11-CEL-PAX4-INS-BLK* [23]. Only 24 (10.1%) were confirmed to have MODY. This study demonstrated that measurable C-peptide during follow-up, family history of diabetes, and a low daily dose of insulin requirement were determined to be the most potential markers to distinguish MODY from T1DM. These characteristics were the same that led us to suspect MODY in our patient.

For beta-cell dysfunction, patients risk being wrongly treated with multiple insulin injections from an early age. It is well known that insulin therapy increases the risk of hypoglycaemia and also brings a lot of psychological issues that deeply affect patients’ quality of life. Since very young, patients treated with insulin have to measure blood glucose levels multiple times per day, undergo a regular medical examination, and organize the injection even when they go out for meals.

For this reason, a selection of the patients based on their clinical features would enable an early diagnosis of MODY, decreasing the risk of severe hypoglycemic episodes, improving patients’ quality of life, and containing the health-care costs related to close self-monitoring of blood glucose. We suggest suspecting MODY and to screen for gene mutation in all patients with a strong family history of diabetes, early-onset, normal BMI, and no autoimmunity.

## Data Availability

The Corresponding Author is available to share the data for future researches, concerning privacy policy.

## Abbreviations

MODY: Maturity onset diabetes of the young
SU: Sulphonylureas
BMI: Body mass index
NGS: Next Generation Sequencing
NEUROD1: Neuronal differentiation 1
SGLT2: Sodium-glucose co-transporter-2
ACMG: American College of Medical Genetics and Genomics
bHLH: Basic helix-loop-helix
